# Biomass and energy potential of *Erianthus arundinaceus* and *Saccharum spontaneum-*derived novel sugarcane hybrids in rainfed environments

**DOI:** 10.1186/s12870-024-04885-0

**Published:** 2024-03-19

**Authors:** Mintu Ram Meena, Perumal Govindaraj, Raja Arun Kumar, Kandasamy Elayaraja, Chinnaswamy Appunu, Ravinder Kumar, Manohar Lal Chhabra, Neeraj Kulshreshtha, Govind Hemaprabha

**Affiliations:** 1 Regional Centre, ICAR– Sugarcane Breeding Institute, Karnal, Haryana 132001 India; 2 ICAR Sugarcane Breeding Institute, Coimbatore-07, Tamil Nadu India; 3 ICAR-CSSRI, Karnal, Haryana 132001 India

**Keywords:** Type-I and Type-II energy canes, Wild genetic resources, Biomass and energy potential, Environment sustainability

## Abstract

**Background:**

Energy canes are viable feedstocks for biomass industries due to their high biomass production potential, lower susceptibility to insects and diseases, better ability to adapt to extreme conditions and clean bioenergy. Interspecific hybrids (ISH) and intergeneric hybrids (IGH) have great potential to meet the growing demand of biomass, biomass-derived energy and feedstock.

**Results:**

In this study, two types of energy canes, Type I and Type II, derived from *S. spontaneum* and *E. arundinaceous* background were evaluated for high biomass, fiber and bioenergy potential under subtropical climate along with the check varieties Co 0238 and CoS 767. Out of 18 energy canes studied, six energy canes, viz., SBIEC11008 (204.15 t/ha), SBIEC11005 (192.93 t/ha), SBIEC13008 (201.26 t/ha), SBIEC13009 (196.58 t/ha), SBIEC13002 (170.15 t/ha), and SBIEC13007 (173.76 t/ha), consistently outperformed the check varieties under Type-I, whereas in type-II, SBIEC11004 (225.78 t/ha), SBIEC11006 (184.89 t/ha), and SBIEC14006 (184.73 t/ha) energy canes produced significantly higher biomass than commercial checks, indicating their superior potential for cogeneration. Estimated energy output from the energy canes (700–1300 GJ/ha/year) exceeded the range of co-varieties (400–500 GJ/ha/year) and energy utilization efficiency in plants and ratoon crops for energy canes viz., SBIEC11008 (3%, 1.97%), SBIEC14006 (1.93%, 2.4%), SBIEC11005 (1.7%, 1.9%), and SBIEC11001 (1.01%, 1.03%), was higher than best checks Co 0238 (0.77, 0.9%). Additionally, energy canes SBIEC 13001 (22.35%), SBIEC 11008 (22.50%), SBIEC 14006 (28.54%), SBIEC 11004 (30.17%) and SBIEC 11001 (27.03%) had higher fiber contents than the co-varieties (12.45%).

**Conclusion:**

The study gives insight about the potential energy canes for higher biomass and energy value. These energy cane presents a vital option to meet the future demand of bioenergy, fiber and fodder for biomass due to their versatile capacity to grow easily under marginal lands without competing with cultivated land worldwide.

**Supplementary Information:**

The online version contains supplementary material available at 10.1186/s12870-024-04885-0.

## Background

Sugarcane, a perennial grass species (*Saccharum spp*.) and an important industrial crop has been utilized over the centuries as a feedstock for sugar production [1]) and in recent decades for ethanol production [2]. However, there is now a growing need to replace fossil fuels and combat the challenge of climate change, which has sparked interest in harnessing sugarcane as an alternative source for energy. The lignocellulosic biomass derived from sugarcane, consisting primarily of bagasse and straw, can be used not only to generate electricity and produce second-generation ethanol but also to create chemicals and bio products in a biorefinery setting [3, 4]. As a result, there has been a shift in the desired traits for sugarcane to meet this growing interest in biomass. The energy cane concept, first introduced by Alexander in 1985 in Puerto Rico, aims to develop sugarcane varieties with greater fiber content and higher biomass yield [5, 6]. The commercial hybrids are primarily created although crossing of two different species, namely, *Saccharum officinarum* and *Saccharum spontaneum. S. officinarum*, known for thick stalks and high sucrose content, constitutes 70–80% of the genome in modern sugarcane cultivars [7]. On the other hand, *S. spontaneum*, which features a thin stalk and high fiber content, accounts for 10–20% of the genome and provides resistance to various diseases and abiotic stressors, as well as increased vigor, hardiness, tillering, and ratooning capabilities [8]. However, energy canes are hybrids between commercial canes and their wild ancestors that have gained popularity due to their high bioenergy feedstock potential. A breeding programme aimed at developing energy cane is achieved through the utilization of high fiber and high biomass-producing *Erianthus arundinaceus* clones with *S. spontaneum* and *S. officinarum* or other commercial cultivars [9]. Enhancing the breeding program for sugarcane cultivars has been crucial in achieving diversification and developing multipurpose varieties [10]. For Type I energycanes, *Saccharum spontaneum* provides high fiber content, more tillers, greater adaptability to adverse climatic conditions, and better ratoonability. A member of the *Saccharum* complex, *Erianthus arundinaceus*, is widely used for developing Type II energy canes since it produces large amounts of biomass as well as its resistance to most pests and diseases. It has been observed that an increase in fiber content is typically accompanied by a reduction in sucrose levels since carbon allocation after photosynthesis is distributed between sucrose storage, respiration, and cell wall biosynthesis [11].

Energy canes have many benefits over commercial sugarcane cultivars as a feedstock for biomass industries due to their high biomass production potential, lower susceptibility to insects and diseases and better adaptation to extreme rainfed conditions. Energy canes have high bioenergy feedstock potential, as they require fewer inputs of water, fertilizers and maintenance over commercial cane varieties. Owing to their higher drought tolerance potential (DTP), energy cane can be easily grown under harsh environmental conditions, such as marginal land and unused land where crops cannot be grown. It is worth noting that the comprehensive review by Kane [12, 13] provides a broader understanding of energy cane as a feedstock for bioenergy production. The review covers various aspects, including biomass characteristics, cultivation practices, processing, and environmental impacts, offering valuable insights into the potential of energy canes as a sustainable bioenergy feedstock. Therefore, utilizing such energy cane in such land can contribute immeasurably to the green energy and sustainability of the ecosystem.

To meet the growing demand for bioenergy, many countries around the world have focused on sugarcane breeding programs for producing energy cane with high fiber content and biomass yield. In the United States, a joint program between Louisiana State University and USDA-ARS achieved a significant increase in biomass yield and 28% fiber content in an energy cane cultivar that continued to increase with each ratoon [14–16] and released several energy cane varieties for use as bioenergy feedstocks, including L 79-1002 [6], Ho 00-961 [17], and Ho 02-113 [18]. The Brazilian company Bio Vertis/Grain Bio has been working on developing energy cane and registered 11 energy canes under the name Vertix® [19, 20]. On the other hand, countries such as India, Argentina, Thailand and Japan have also reported progress in improving energy cane varieties. In the energy breeding programs at the ICAR-Sugarcane Breeding Institute, Coimbatore, India, many energy canes are developed under the series “SBIEC” (Sugarcane Breeding Institute Energy canes). These SBIEC series consist of two types of energy canes: type-I and type-II energy canes [21]. The Type I energy cane has a juice brix higher than 15% and a fiber higher than 20%, while the Type II energy cane has a fiber higher than 25% and a juice brix higher than 10%. Type-I energy canes are dual purpose energy canes because the juice can be used for direct fermentation in distilleries to produce alcohol, and the bagasse can be used in cogeneration units to generate electricity. For Type I energy canes, *Saccharum spontaneum* provides high fiber content, more tillers, greater adaptability to adverse climatic conditions, and better ratoonability [9]. A member of the *Saccharum complex, Erianthus arundinaceus*, is widely used for developing Type II energy canes since it produces large amounts of biomass as well as its resistance to most pests and diseases. In view of this, a total of 18 energy canes consisting of types I and II derived from wild species of Saccharum and E. arundinaceous were developed and evaluated for their high biomass, fiber%, ratooning potential and other stress tolerance traits under rainfed marginal land of subtropical climates. In numerous breeding programs, energy cane has been shown to have a higher biomass potential than sorghum, elephant grass, and eucalyptus. The breeding program at the ICAR-Sugarcane Breeding Institute has led to the development of high biomass-producing energy cane utilizing *S. spontaneum* and *Erianthus arundinaceous* back ground into cultivated sugarcane lines [22, 23].

The current study on recently developed energy canes reported a great economic advantage over the check varieties to produce first- or second-generation ethanol.

## Methods

### Experimental site

The experiment was planted at the ICAR-Sugarcane Breeding Institute, Regional Centre, Karnal (Haryana) India, which is located in 29.1°–29.5° N and 76.3°–77.1° E in a subtropical climate, with an elevation of 243 m above mean sea level and an average annual rainfall of approximately 744 mm. In summer, the maximum temperature ranges from 34 to 45 °C, while in winter, the minimum temperature ranges from 5 to 8 °C. The soil in this area varies from clay-loamy to loam, with a pH range of 8.0–8.5.

### Plant material and experimental design

A total of eighteen clones consisting of fourteen clones of the type-I energy canes and four clones of the type-II energy canes were evaluated for their biomass contributing traits and energy value along with two standard sugarcane varieties, i.e., Co 0238 and CoS 767. Type-I energy canes are *S. spontaneum*-based energycanes viz., SBI EC 11005, SBI EC 11002, SBI EC 11003, SBI EC 11008, SBI EC 11009, SBI EC 13001, SBI EC 13002, SBI EC 13005, SBI EC 13007, SBI EC 13008, SBI EC 13009, SBI EC 13010, SBI EC 14003 and under type-II energy canes, are *E. arundinaceus*-based energycanes, viz., SBI EC 11001, SBI EC 11004, SBI EC 11006 and SBI EC 14006 (Table 1). The date of planting for the plant crop was in the first week of March during 2017-18 crop season, and harvested during March-April, 2018-19. Similarly, the ratoon crop was raised in 2018-19 during the spring season (Feb-March). The experiment was carried out over a span of two years (2017–18 and 2018–19 spring seasons) using a randomized block design with three replications. Plots utilized had dimensions of 2 m × 6 m and were spaced 0.9 m apart. Standard recommended practices were followed for crop production. Three lifesaving irrigations were applied at the time of germination, tillering and grand growth stage of the crop; otherwise, the crop was managed upon natural rainfall throughout the year. The observations were collected from each plot, specifically for the number of millable canes (NMC), brix% in cane, single cane weight (SCW), dry cane weight (DCW), total dry weight (TDM), and fresh biomass weight (FBW). HR Brix, pol % in juice, single cane weight, cane height, cane diameter, biomass yield and energy were estimated from 10-month-old crop. At a crop age of 10 months, five canes randomly selected from each plot were tagged and assessed. For the estimation of fiber percentage, 250 g subsamples of shredded canes were crushed in a rapipol machine and subsequently oven dried. The fresh and dry weights of these samples were recorded. Fiber percentage was determined using rapipol extraction method, as outlined by Thangavelu and Rao [24].

**Table 1:** Types of energy cane clones along with their parentages

Type of energycane	Clone	Clone name	Parentage
Type I	SBIEC 11002	SSCD 682	Co 1148 x SES 404 (2n = 64)
Type I	SBIEC 11003	SSCD 1013	Co 8371 x SH 216 (2n = 72)
Type I	SBIEC 11005	SSCD 941	Co 8371 x SES 574 (2n = 80)
Type I	SBIEC 11008	SSCD 849	Co 8371 x SES 410 (2n = 64)
Type I	SBIEC 11009	IA 1167	Co cane x *S. spontaneum*
Type I	SBIEC 13001	BM 10,192	IGH 037801 x CoJ 03193
Type I	SBIEC 13002	BM 10,135	IGH 013504 x CoJ 03193
Type I	SBIEC 13005	BM 10,161	Co 8471 x IND 84–415
Type I	SBIEC 13007	BM 10,164	Co 8471 x IND 84–415
Type I	SBIEC 13008	BM 10,286	IK76-92 x 98 N1 1405
Type I	SBIEC 13009	BM 10,110	IGH 013504 x Co 0218
Type I	SBIEC 13010	BM 10,122	MS 6847 x IND 87–145
Type I	SBIEC 14002	BM 10,177	IGH 038701 x Co 62,198
Type I	SBIEC 14003	BM 10,184	ISH 100 x PL 480 − 376
Type II	SBIEC 11004	IK 76–92 × 98 N1 1405	IK 76–92 x 98 N1 1405
Type II	SBIEC 14006	BM 09283	IK 76 − 75 GC
Type II	SBIEC 11006	IK 76–92 × 98 N1 1405	IK 76–92 x 98 N1 1405
Type II	SBIEC 11001	IK 76–92 × 98 N1 1405	IK 76–92 x 98 N1 1405

*Note* *Type I are *S. spontaneum* based energycanes and **type-II** are *E. arundinaceus* based energycanes.

$$\text{Fiber percent} = (\text{A}-\text{B})/\text{C} \times 100$$

in the given context, A represents the dry weight of bagasse (residue) plus the bag after the drying process (measured in grams). B represents the dry weight of the bag alone (measured in grams). C represents the fresh weight of the cane (measured in grams) [25].

To calculate the dry matter percentage in cane, brix percentage in cane, and dry biomass yield, the following formulas were applied:

$$\begin{aligned} & \text {Dry Matter Percentage in Cane} = \\ & \quad \frac{\left(\text{WSB}-\text{WSA}\right)* \text{Brix in}\frac{Juice}{100}+WSA*\frac{DMB}{100})}{WSB}*100 \end{aligned}$$

In this formula, WSB represents the weight of the sample before crushing (measured in grams). WSA represents the weight of the sample after crushing, which refers to the bagasse (measured in grams). Brix in Juice represents the brix percentage in the cane. By substituting the respective values into the formula, the dry matter percentage in cane can be calculated.

To compute the fresh biomass yield (measured in tons per hectare, t/ha), the formula used was

$$\begin{aligned} & \text{fresh biomass yield} = \\ & \quad NMC-ha \, X \, Scwt \left(kg\right) with \; cane\; top \end{aligned}$$

Additionally, the dry biomass yield (also measured in tons per hectare, t/ha) was determined using the following formula as described by Mohanraj et al. [26]:

$$\begin{aligned} & \text{Dry Biomass Yield} = \\ & \quad \text{Dry Matter Percentage} \times \text{Fresh Biomass Yield} \end{aligned}$$

### Energy calculation

The energy content of plant biomass is primarily influenced by its composition, with fats and proteins having higher energy contents than simple carbohydrates. Sugarcane, being primarily composed of carbohydrates such as sugar and lignocellulose, possesses an energy content of approximately 15.9 MJ/kg. To determine the energy content, the total dry biomass was multiplied by 15.9 and expressed as gigajoule per hectare per year (GJ/ha/year) [27].

### Red rot evaluation

To assess their resistance to red rot, a comprehensive screening process was conducted on all type-I and type-II energy canes. This study aimed to evaluate their resistance against the predominant and highly virulent pathotypes of red rot, *Colletotrichum falcatum*, specifically the CF 08 and CF 09 races [28]. The screening was performed under field conditions. To initiate the screening, a mixed inoculum containing both races of *Colletotrichum falcatum* was prepared. This inoculum was then applied to the sugarcane plants through plug and nodal methods. The screening was conducted when the crops were approximately seven months old, with inoculation occurring in September.

### Statistical analysis

The collected data underwent analysis of variance (ANOVA) tests for statistical evaluation. The statistical package SAS 9.3 software (SAS Institute Inc., Cary, USA) was utilized to calculate means, standard deviations, and coefficients of variance for various traits. The remaining data were analyzed using the R platform.

### Confirmation of intergeneric hybrids

*Erianthus*-specific markers were developed from 5 S rRNA spacer regions using sequence tagged PCR. *Erianthus arundinaceus* clone IK 76–81 along with randomly selected intergenic hybrids and *Saccharum spp.* hybrid Co 86,032 (commercial variety) were screened with primers that amplify 5 S rDNA regions in the genome to identify *Erianthus*-specific fragments. PCR amplification conditions for 5 s rDNA [29] were followed. Amplified products were resolved on 2% agarose gels stained with ethidium bromide and documented using a gel documentation system (Syngene, USA).

## Results

### Analysis of variance

A high degree of variance was observed for all traits in both years among the clones under study. Two-way analysis of variance indicated that there was a significant difference among the type-I and type-II energy clones with respect to the biomass yield and quality traits in both years (Table 2). The interaction term for year and clone (year × clone) identity was significant for all traits, which indicates that the performance of the clones varied between the two years of crop. Furthermore, the interaction between year and replication was found to be nonsignificant, indicating that replications over different years do not have a significant impact on the traits studied.

**Table 2:** Results of the two-way analysis (F-ratio) showing the effects of genotypes and year on the different traits of Energy clones

Source	F-ratio and significance								
	D. f.	NMC	SCWT	FBMY	DBY	Fibre%	Brix%	Pol%	GJ/ha/yr
Year	1	89.54***	17.08***	118.37***	68.09***	68.09***	298.74***	89.02***	1.31ns
Clone	19	2.28*	6.57***	1.64ns	4.96**	4.96***	2.43*	3.19**	1.70ns
Rep*year	2	0.48ns	0.58ns	0.73ns	0.73ns	0.73ns	0.16ns	0.42ns	1.57ns
Clone*year	19	18.85***	11.64***	21.38***	10.39***	10.39***	64.42***	34.69***	14.58***
Error	38								

SCW: single cane weight, NMC: number of millable canes, TDM: total dry matter, DM: dry matter, FBMY: fresh biomass yield, GJ: Gigajoule).*** denotes *p* < 0.001, ** denotes *p* < 0.01; * denotes *p* < 0.05; and ns denotes non-significant *p* ≥ 0.05.

### Enhanced total biomass production in type-1 and type-II energy canes

A notable distinction was observed among the energy canes in terms of fresh biomass yield and dry biomass. Average fresh biomass yield of type-I energy cane was 156.35 t/ha, where type-II energy canes recorded a yield of 167.06 t/ha which was significantly higher than in contrast to the commercial cultivated sugarcane varieties (107.58 t/ha) in the plant crop. This indicates a significant improvement of 31.19% and 35.60% in fresh biomass yield for type-I and type-II energy canes, respectively, compared to commercial canes. In the ratoon crop, the mean fresh biomass yield for type-I energy cane was 164.98 t/ha, while type-II energy cane yielded 189.96 t/ha. These energy canes showed improvements of 14.23% and 25.52%, respectively, in fresh biomass yield compared to commercial sugarcane varieties. Among the different clones evaluated, six clones, namely, SBIEC11008 (204.15 t/ha), SBIEC11005 (192.93 t/ha), SBIEC13008 (201.26 t/ha), SBIEC13009 (196.58 t/ha), SBIEC13002 (170.15 t/ha), and SBIEC13007 (173.76 t/ha), demonstrated significantly higher fresh biomass yields under type-I energy canes. Of these, SBIEC11005 (198.07 t/ha) and SBIEC11008 (220.77 t/ha) consistently outperformed the check varieties in both plant and ratoon crops. Within the type-II energy cane category, two energy canes stood out in the plant crop: SBIEC11004 (159.11 t/ha) and SBIEC (236.62 t/ha). In the ratoon crop, three energy canes, SBIEC11004 (225.78 t/ha), SBIEC11006 (184.89 t/ha), and SBIEC14006 (184.73 t/ha), exhibited significantly better biomass yields than the commercial check varieties. These findings highlight the potential of these energy canes to meet future demands for biomass feedstock and bioenergy.

**Fig. 1:**
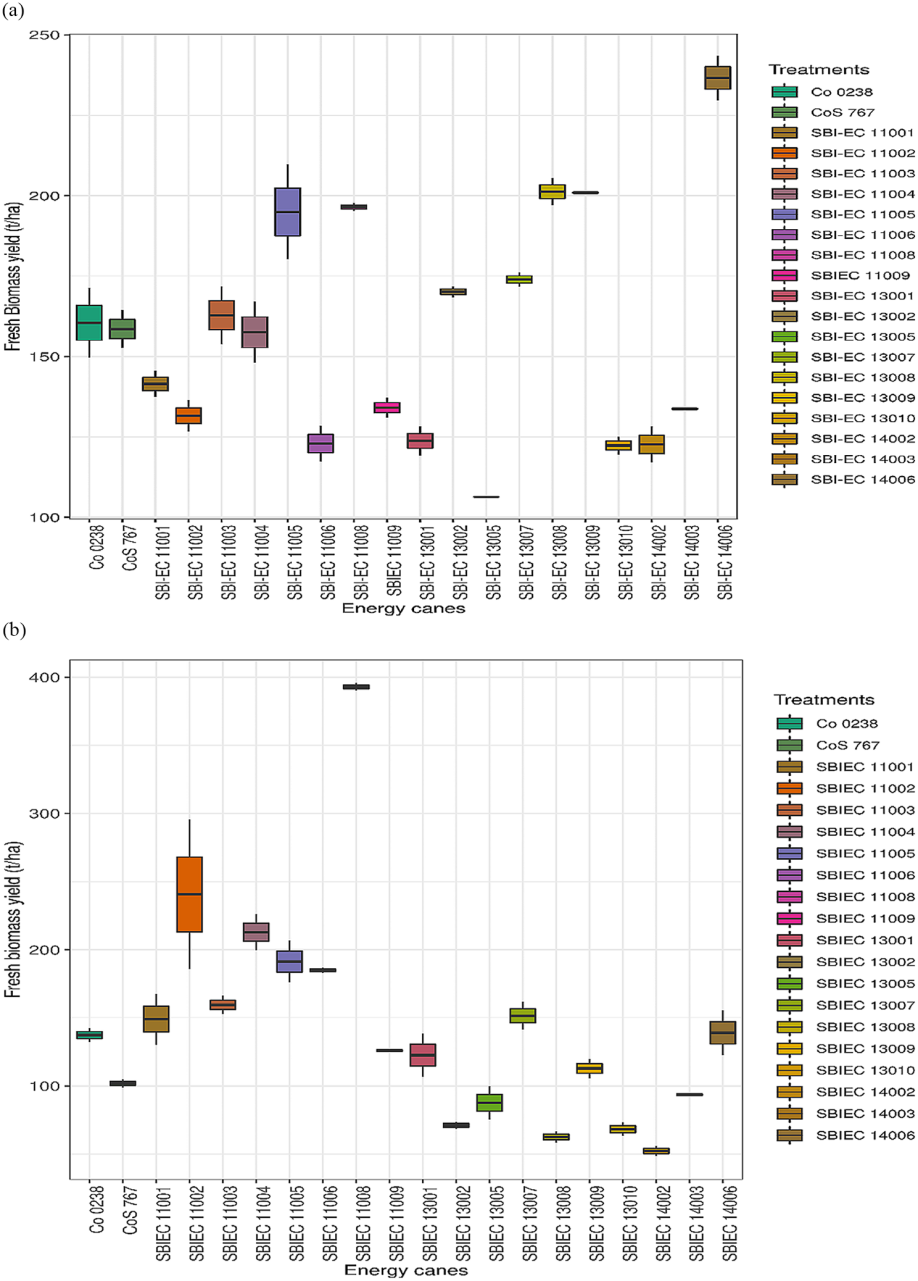
Fresh biomass yield (t/ha) obtained from type-I and type-II energy canes. **a** Fresh biomass yield (t/ha) obtained from type-I and type-II energy canes (Plant Crop). **b** Fresh biomass yield (t/ha) obtained from type-I and type-II energy canes (Ratoon Crop)

Similarly, the dry biomass potential of type-I and type-II energy canes was assessed at harvest stages. The average dry biomass yield for type-I energy cane was 38.20 t/ha in the plant crop and 43.63 t/ha in the ratoon crop. Type-II energy cane yielded 42.64 t/ha in the plant crop and 49.58 t/ha in the ratoon crop. In comparison, commercial cane varieties produced 35.48 t/ha and 36.26 t/ha of dry biomass in the respective crops (Table 3; Fig. 1). The total dry biomass percent in type-I energy canes recorded was 24.45%, whereas it was 28.66% in type-II energy canes. Both types of energy canes had higher TDM% than the commercial canes (22.22%). The confirmation of these *S. spontaneum-* and *E. arundinaceous*-derived hybrids with commercial sugarcane is presented in Fig. S2.

**Table 3:** Total Biomass yield and its contributing traits in Type-I and Type-II energy canes

Clones name	NMC/ha		SCWT		Cane dia		Cane Ht		Cane top wt		TDM (%) in cane		Cane yd (t/ha)		FBMY (t/ha)		Fibre (%)		TDM (t/ha)	
	Plant	Ratoon	Plant	Ratoon	Plant	Ratoon	Plant	Ratoon	Plant	Ratoon	Plant	Ratoon	Plant	Ratoon	Plant	Ratoon	Plant	Ratoon	Plant	Ratoon
**Type-I energy canes**																				
SBIEC 11002	116049.38	167870.37	0.81	0.90	1.80	1.8	175.0	265	0.42	0.67	23.87	28.69	97.37	150.81	142.16	263.56	19.55	19.91	31.43	69.17
SBIEC 11003	117283.95	155555.56	0.91	0.52	1.55	1.6	241.7	275	0.61	0.46	22.93	29.85	96.09	81.63	162.83	159.48	19.55	17.54	37.38	47.68
SBIEC 11005	117283.95	148703.70	0.84	0.63	1.55	1.5	185.0	300	0.81	0.70	26.00	39.38	98.04	94.30	192.93	198.07	22.71	22.75	50.46	75.24
SBIEC 11008	130864.20	135444.44	0.83	0.80	1.85	1.9	336.7	302.5	0.74	0.83	24.00	30.15	106.93	164.66	204.15	220.77	22.50	22.24	47.15	118.4
SBIEC 11009	132098.77	113333.33	0.53	0.42	1.57	1.6	232.0	230	0.54	0.70	26.34	35.08	66.11	46.55	140.69	126.54	22.42	20.21	35.30	44.21
SBIEC 13001	134567.90	140740.74	0.65	0.47	1.20	1.3	241.7	250	0.31	0.48	24.06	33.86	84.83	37.13	128.51	133.56	22.35	22.80	29.80	41.71
SBIEC 13002	151851.85	155555.56	0.56	0.46	1.40	1.5	231.0	215	0.59	0.38	29.18	34.22	87.31	40.01	170.15	171.08	22.93	23.34	49.68	24.29
SBIEC 13005	93827.16	99629.63	0.66	0.53	1.60	1.6	165.0	225	0.49	0.57	23.54	31.15	61.85	41.84	107.76	109.29	19.63	18.30	25.04	26.90
SBIEC 13007	104938.27	98888.89	0.93	0.94	1.90	2.0	140.3	230	0.88	0.61	20.48	27.45	97.78	83.79	173.96	153.18	14.17	11.29	35.62	41.73
SBIEC 13008	141975.31	114814.81	0.56	0.58	1.65	1.5	195.0	167.5	0.88	0.69	24.15	30.18	79.09	18.07	201.26	162.55	19.91	17.73	48.57	18.77
SBIEC 13009	166666.67	158370.37	0.55	0.42	1.45	1.6	242.0	240	0.63	0.62	28.45	28.62	92.00	45.41	196.58	164.55	22.22	23.80	57.16	32.30
SBIEC 13010	96296.30	92777.78	0.83	0.71	1.30	1.2	183.7	235	0.44	0.42	28.43	33.32	79.93	47.59	122.34	128.33	22.71	21.45	34.79	22.75
SBIEC 14002	146913.58	139629.63	0.69	0.63	2.00	1.9	311.7	275	0.14	0.20	20.81	22.96	98.81	34.01	122.67	152.31	14.38	13.83	25.47	11.99
SBIEC 14003	112345.68	114814.81	0.96	0.91	1.40	1.5	195.0	240	0.13	0.54	20.13	23.32	112.00	58.96	122.91	166.48	17.45	15.10	26.92	21.78
Mean	125925.93	131152.12	0.73	0.64	1.59	1.6	219.69	246.4	0.54	0.56	24.45	30.59	89.87	67.48	156.35	164.98	20.17	19.31	38.20	42.64
**Type-II energy canes**																				
SBIEC 11001	128395.06	128888.89	0.49	0.49	1.65	1.4	335.0	300	0.68	0.79	28.64	29.07	64.67	52.50	149.58	164.46	27.03	26.68	40.54	43.23
SBIEC 11004	177777.78	267037.04	0.49	0.32	1.36	1.4	233.3	245	0.41	0.53	32.19	33.53	84.56	57.55	159.11	225.78	30.17	29.90	33.39	71.60
SBIEC 11006	130864.20	160370.37	0.46	0.42	1.80	1.9	285.0	240	0.47	0.61	23.41	26.03	58.85	67.68	122.93	184.89	21.75	22.59	28.77	48.14
SBIEC 14006	177777.78	180925.93	0.82	0.53	1.60	1.5	278.3	275	0.52	0.49	30.40	25.42	143.31	80.02	236.62	184.73	28.54	27.61	71.82	35.37
Mean	153703.70	184305.56	0.56	0.44	1.60	1.5	282.92	265.0	0.51	0.54	28.66	28.51	87.85	64.44	167.06	189.96	26.87	26.70	43.63	49.58
**Commercial cane varieties (Checks)**																				
Co 0238	72222.22	84987.65	1.09	1.07	2.30	2.4	225.0	214.5	0.64	0.74	23.39	30.77	100.57	77.33	125.16	153.82	12.94	12.33	37.50	42.25
CoS 767	84185.19	96913.58	0.66	0.85	1.90	1.9	218.3	212.5	0.42	0.48	21.05	29.71	94.48	71.55	90.00	128.52	12.65	12.56	33.36	30.26
Mean	78203.70	90950.62	0.87	0.96	2.10	2.2	221.67	213.5	0.54	0.57	22.22	30.24	97.53	74.44	107.58	141.49	12.79	12.44	35.43	36.26
GM	127900.67	126987.96	0.71	0.66	1.64	1.65	234.2	250	0.53	0.58	25.21	30.14	90.10	67.57	153.62	142.88	21.02	17.45	39.18	43.39
LSD @ 5%	14966.00	14364.95	0.10	0.12	0.36	0.37	66.0	59.68	0.62	0.53	2.74	4.18	14.62	21.29	42.84	45.60	2.86	1.72	11.97	14.87

SCW: single cane weight, NMC: number of millable canes, TDM: total dry matter, TDM: total dry matter, FBMY: fresh biomass yield.

### Energy utilization efficiency of energy canes

**Fig. 2:**
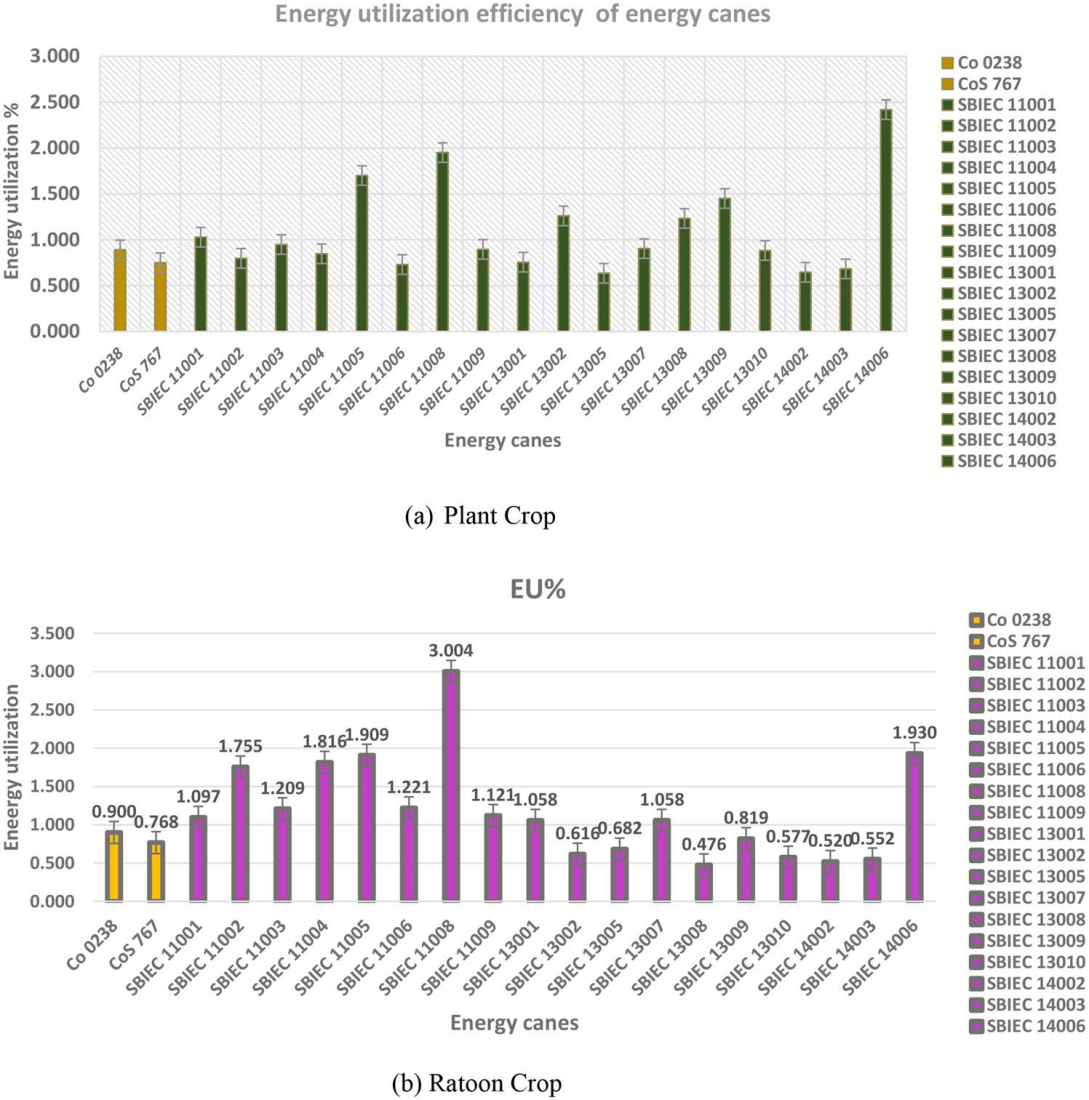
Energy utilization efficiency of energy canes. **a** Energy utilization efficiency of energy canes (Plant Crop). **b** Energy utilization efficiency of energy canes (Ratoon Crop)

In this study, the energy utilization efficiency of energy canes ranged from 1 to 3%. In plant crops and ratoon crops, there were seven energy canes and nine energy canes, respectively, that exhibited higher energy utilization efficiency compared to the best check variety Co 0238 (0.95%). The top-ranking clones for energy utilization in the ratoon and plant crop were SBIEC11008 (3%, 1.97%), SBIEC14006 (1.93%, 2.4%), SBIEC11005 (1.7%, 1.9%), and SBIEC11001 (1.01%, 1.03%). Among these clones, SBIEC14006 and SBIEC11001 belong to the type-II energy canes category, while SBIEC11008 and SBIEC11005 are type-I energy canes (Fig. 2).

### Cane growth and quality parameters

The average cane height at the harvest stage for type-I and type-II energy canes was recorded as 246 and 282 cm, respectively, which exceeded the height of sugarcane varieties (221 cm). Notably, the energy canes SBIEC 11008 (336 cm), SBIEC11001 (335 cm), SBIEC 11005 (302 cm) and SBIEC 14006 (278 cm) exhibited the tallest cane heights, indicating their superior potential under rainfed conditions. Likewise, the individual weight and top cane weight in energy canes varied between 0.49 and 0.96 kg and 0.14 and 0.88 kg, respectively, which were comparable to those observed in commercial sugarcane varieties. The cane diameter in the energy canes was slightly lower than that in the cultivated canes. However, certain energy canes, such as SBIEC 14002 (2.0 cm), SBIEC 11006 (1.9 cm), and SBIEC 13007 (1.95 cm), exhibited cane diameters that were on par with sugarcane varieties. The number of millable canes (NMC) in energy canes recorded was higher than that of sugarcane varieties. The average number of millable canes (NMC) in type-I energy canes ranged from 0.93 lakhs to 1.70 lakhs per hectare, while in type-II energy canes, it ranged from 1.12 lakhs to 1.77 lakhs per hectare.

The average brix percentage in type-I and type-II energy canes was recorded as 17.0% and 9.37%, respectively, while it was 18.75% in commercially cultivated canes. Therefore, type-I energy canes can be utilized to obtain relatively lower sugar content compared to commercial canes while still achieving higher biomass yield. On the other hand, type-II energy canes are primarily suitable for generating higher biomass due to their higher fiber percentage. Type-I energy canes in the study with comparable pol% (percentage of sucrose) in juice were observed in SBIEC 14003 (17.12%), SBIEC 11005 (16.09%), SBIEC 13008 (16.50%), and SBIEC 13007 (16.40%). By categorizing energy canes into Type I and Type II based on their specific characteristics, such as juice brix and cane fiber content, they can be effectively utilized for different purposes, with Type I primarily focused on alcohol production and Type II on electricity generation. (Table S1).

### Exploring high fiber content in energy canes

The fiber percentage and industrial utilization of the energy canes were also assessed. The fiber content varied among the different energy canes. The energy canes had higher fiber percentages (20–28%) than other commercial canes (10–12%). The high fibrous biomass available in energy canes can be converted into biofuels and used as a renewable energy source. The average fiber percentages in type-I energy cane in the ratoon crop and plant crop were 19.31% and 20.17%, respectively. In contrast, the average fiber percentage in type-II energy cane was 26.87% in the plant crop and 26.70% in the ratoon crop. A higher fiber percentage was recorded in type-II clones SBIEC14006 (28.54%), SBIEC11004 (30.17%) and SBIEC11001 (27.03%), indicating that their proportion of fiber content could be processed to produce fiber suitable for weaving into textiles, such as bags, clothing, and home textiles. These variations indicated that certain canes had a higher proportion of fiber content, making them more suitable for industrial applications [30]. Cane with high fiber content can be utilized in the production of biodegradable materials, such as bioplastics and packaging, offering sustainable alternatives to traditional plastic products.

### Energy potential of energy canes

**Fig. 3:**
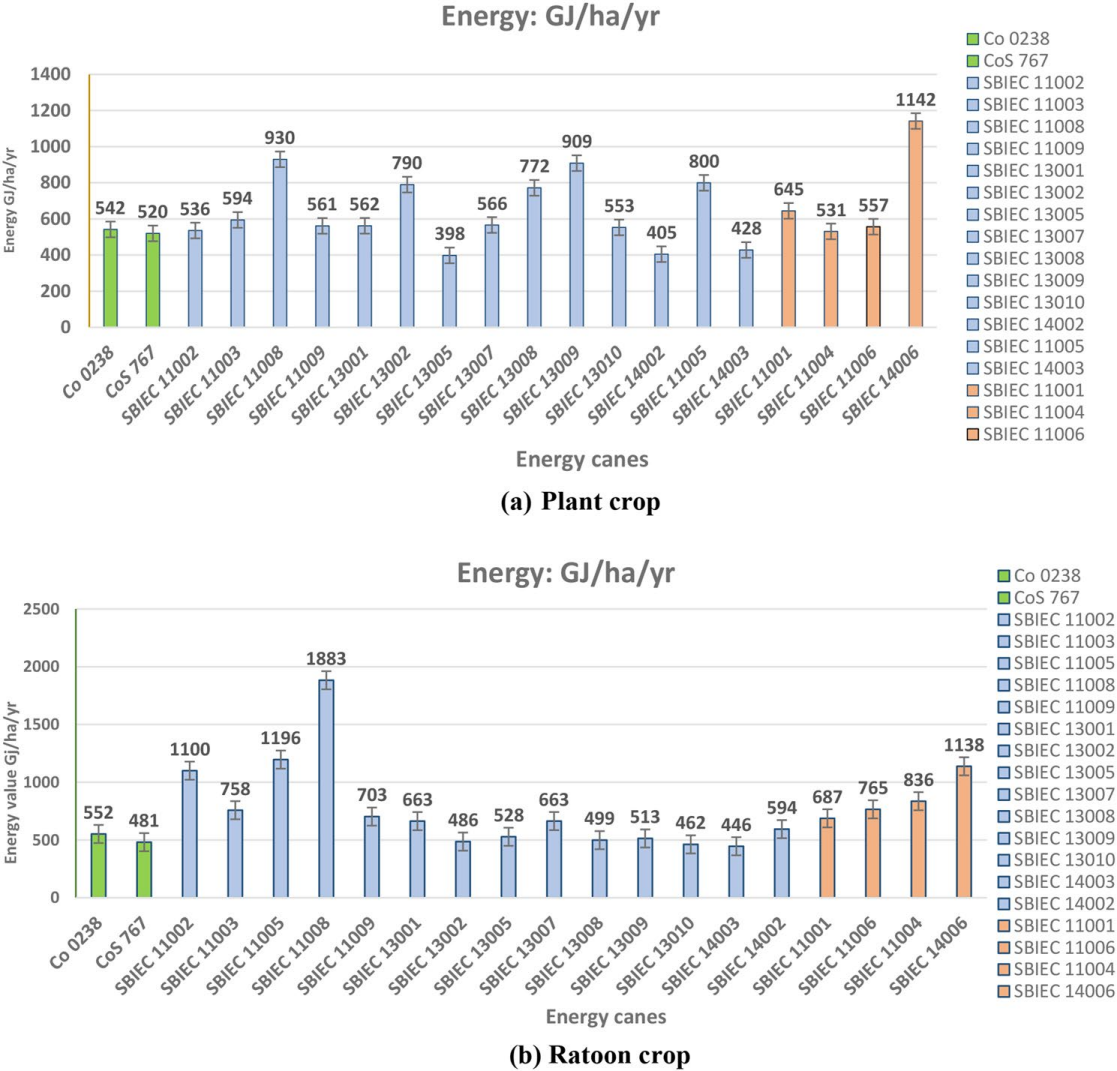
Energy potential of type-I and type-II energy clones vs. sugarcane commercial checks. **a** Energy potential of type-I and type-II energy clones vs. sugarcane commercial checks (Plant crop). **b** Energy potential of type-I and type-II energy clones vs. sugarcane commercial checks (Ratoon crop)

The average energy production from sugarcane is approximately 400–500 GJ/ha/year. However, in type-I energy canes, the range of energy production is 530–930 GJ/ha/year, while in type-II energy canes, it varies from 451 to 1142 GJ/ha/year. These findings highlight the significantly higher energy potential of energy canes compared to commercial canes. This increased energy potential positions energy canes as suitable sources for the production of second-generation ethanol, especially considering the growing demand to meet the future needs of a growing population. Among the Type-I energy canes, the top-ranking clones for energy potential were SBIEC 11008 (930 Gj/ha/year), SBIEC 13009 (909 GJ/ha/year), SBIEC 11005 (800 GJ/ha/year), SBIEC 13002 (790 GJ/ha/year) and SBIEC 13008 (772 GJ/ha/year). These clones demonstrated superior energy potential compared to others in this category. In the case of Type-II energy canes, SBIEC 14006 (1142 GJ/ha/year) and SBIEC 11001 (645 GJ/ha/year) exhibited higher energy potential compared to the rest of the clones. These two energy canes displayed exceptional capabilities for energy production within the Type-II category. Similarly, the energy value for ratoon crops was estimated, and SBIEC 11008 (11,883 GJ/ha/year), SBIEC 11005 (1196 GJ/ha/year), SBIEC 14006 (1138 GJ/ha/year) and SBIEC 11002 (1100 GJ/ha/year) produced higher energy values [31] (Fig. 3). Hence, these mentioned energy canes might be a good source of biomass energy.

### Estimation of harvest index from energy canes

**Fig. 4:**
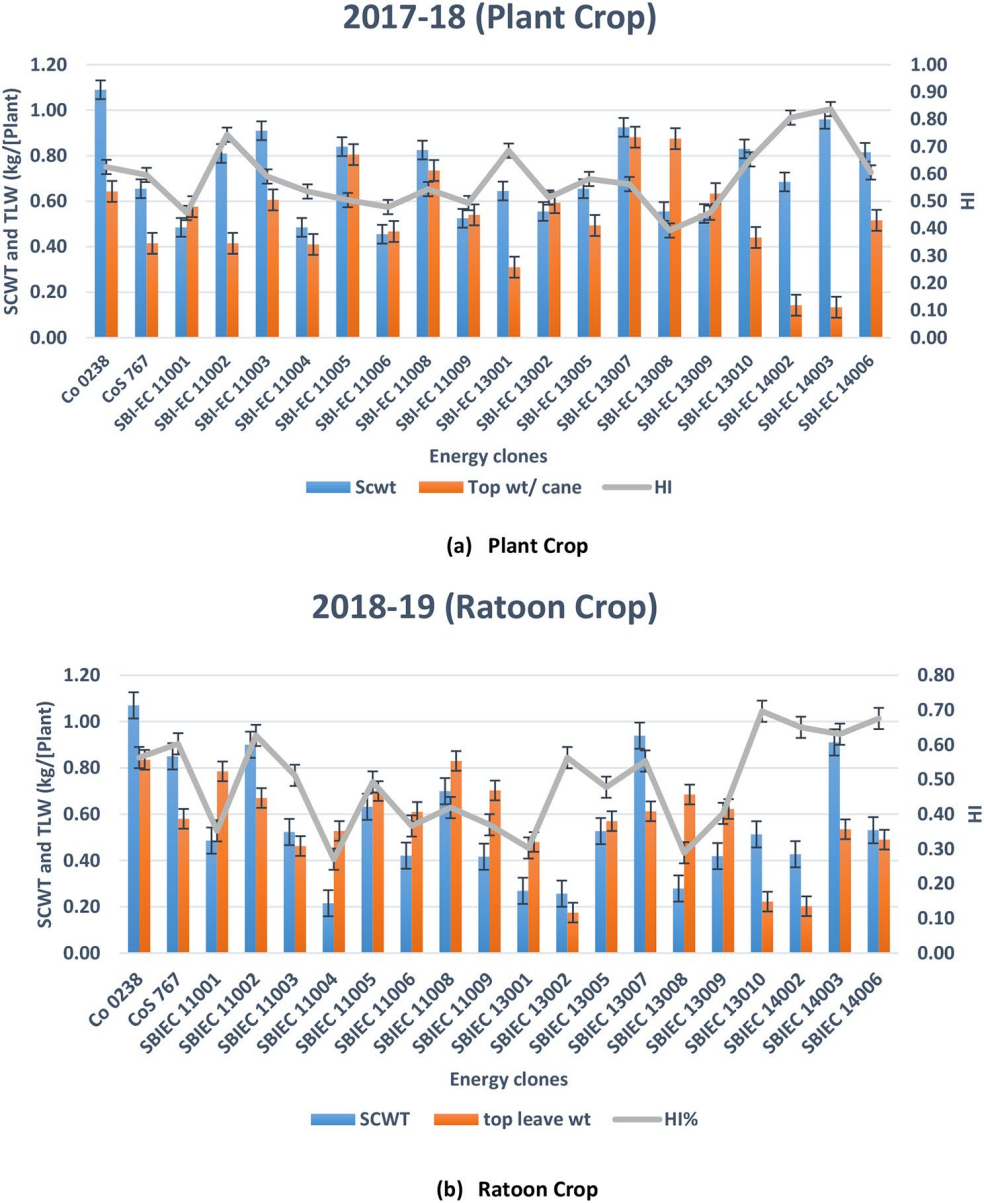
Estimation of Harvest Index from energy canes. **a** Estimation of Harvest Index from energy canes (Plant Crop), **b** Estimation of Harvest Index from energy canes (Ratoon Crop)

The harvest index (HI) is an important parameter in sugarcane cultivation. It is a measure of the efficiency with which a plant allocates energy and resources toward the harvested product, which in this case are the sugarcane stalks. The HI is calculated by dividing the weight of the harvested product (sugarcane stalks) by the total aboveground biomass of the plant. The harvest index (HI) was determined for energy canes at the 10th month of both the plant crop and ratoon crop stages. The average HI for commercial canes typically ranges from 0.59 to 0.6. In the case of energy canes, the HI varied between 0.59 and 0.84. Among the energy cane varieties, type-I energy canes, specifically SBIEC 14002, exhibited the highest HI of 0.81, followed by SBIEC 11002 with an HI of 0.74. On the other hand, type-II energy canes, specifically SBIEC 14003, had the highest HI of 0.84, while SBIEC 14006 recorded an HI of 0.61. In general, energy canes tend to have higher harvest indexes than sugarcane. This is because energy canes are selected and bred to allocate a larger proportion of their biomass toward the harvested product, which is usually used for biofuel or bioenergy purposes. (Fig. 4).

### Correlation matrix of biomass contributing traits in energy canes

A correlation analysis was conducted to examine the relationships between biomass yield and its contributing traits. The results revealed significant correlations between various variables. The correlation coefficients indicate the direction and strength of these relationships. Noteworthy findings include a strong positive correlation of 0.933 between fresh biomass yield (FBM) and total dry matter (TDM). Additionally, cane yield and TDM exhibited a moderate positive correlation of 0.65, while a significant positive correlation of 0.80 was observed between fresh biomass yield and cane yield. Moreover, cane volume displayed a moderately positive correlation of 0.72 with TDM, and a similar correlation of 0.71 was found between cane volume and FBM. Last, the correlation between cane volume and cane yield was 0.58, indicating a positive association between these variables. These correlation coefficients provide insights into the relationships between biomass yield and its contributing traits (Figure S1).

### Resistance of energy canes against the red rot

A total of eighteen energy cane clones were subjected to the screening process. The results of the screening revealed varying levels of resistance among the tested clones. A high level of resistance (R) to red rot against virulent pathotypes CF08 (5R) and CF09 (8R) was observed, indicating their ability to withstand infection and exhibit minimal disease symptoms. The clones were classified as moderately resistant (MR), indicating a moderate level of resistance to the pathogen. On the other hand, the clones were categorized as moderately susceptible (MS), indicating a higher susceptibility to red rot compared to the resistant and moderately resistant clones. Last, clones that are classified as susceptible (S) indicate their vulnerability to infection and the development of severe disease symptoms (Table 4).

**Table 4:** Red rot evaluation of energy canes against the prevailing virulent pathotypes

Clone Name	Red rot rating (CF 08)	Red rot rating (CF 09)
Type-I Energy Canes		
SBIEC 11,002	S	MS
SBIEC 11,003	MR	R
SBIEC 11,005	MR	MR
SBIEC 11,008	R	R
SBIEC 11,009	S	MS
SBIEC 13,001	S	MS
SBIEC 13,002	S	S
SBIEC 13,005	R	R
SBIEC 13,007	MR	R
SBIEC 13,008	MR	MR
SBIEC 13,009	HS	S
SBIEC 13,010	MS	MR
SBIEC 14,002	S	MS
SBIEC 14,003	HS	S
**Type-II Energy Canes**		
SBIEC 11,006	R	R
SBIEC 11,004	R	R
SBIEC 11,001	R	R
SBIEC 14,006	MR	R
**Standard checks**		
Co 0238	R	R
CoS 767	MS	MR

R: Resistant, MR: Moderately resistant, MS: Moderately Susceptible,

HS: Highly Susceptible, S: Susceptible.

## Discussion

Energy canes, which are specific hybrids of Saccharum species along with allied genera, have gained significant attention as a dedicated biomass crop for bioenergy production. They are selected and bred to maximize biomass yield, making them suitable for various bioenergy applications. Several studies have been conducted to investigate different aspects of energy canes and their potential as a feedstock for bioenergy. Our study has revealed the significant biomass yield potential of numerous energy canes belonging to both Type-I and Type-II categories. These energy canes hold great promise for utilization in industries due to their abundant biomass production, making them valuable for bioenergy purposes. Moreover, a notable advantage of these energy canes is their adaptability to subtropical climates, where they can be successfully cultivated under rainfed conditions. This suggests that energy cane cultivation could be a viable option for sustainable bioenergy production in regions with similar climatic characteristics. The average fresh biomass yield for type-I energy cane was 156.35 t/ha, while type-II energy cane recorded a yield of 167.06 t/ha. In contrast, the commercial cultivated sugarcane varieties yielded 107.58 t/ha in the plant crop. This indicates a significant improvement of 31.19% and 35.60% in fresh biomass yield for type-I and type-II energy canes. These findings highlight the superior biomass productivity of energy canes, particularly in the case of type-II varieties, surpassing the yields achieved by conventional sugarcane varieties due to their higher carbon partitioning [32]. Past research [33, 34] has demonstrated a substantial increase in biomass yield in energy cane varieties, with the partitioning of carbon and energy being focused on the harvested product. High biomass potential in interspecific hybrids (ISH) was also studied, and some ISH hybrid canes with high biomass and energy were found [35, 36]. These findings suggest that energy canes can effectively allocate resources toward biomass production, making them a promising feedstock for bioenergy [37, 38].

A significantly higher energy potential was observed for energy canes compared to commercial canes. The increased energy potential positions energy canes as suitable sources for the production of second-generation ethanol [39] especially considering the growing demand to meet the future needs of a growing population. Based on our findings, certain energy cane varieties exhibited higher energy potential compared to other clones in both Type-I and Type-II categories. Among the Type-I energy canes, SBIEC 11008, SBIEC 13009, SBIEC 11005, SBIEC 13002, and SBIEC 13008 demonstrated superior energy potential. Similarly, in the Type-II energy canes, SBIEC 14006 and SBIEC 11001 showcased higher energy potential. Vilarinho investigated the biomass yield and energy potential of energy canes grown in Southern Europe, highlighting their adaptability to different climates [40]. evaluated the productivity and energy potential of energy cane varieties in Brazil, emphasizing the importance of selecting suitable cultivars for specific regions. These results suggest that these specific energy cane varieties have promising attributes for efficient energy production compared to the other clones studied [41]. The theoretical maximum limit for energy utilization in plants is determined by the efficiency of photosynthesis, the process by which plants convert sunlight into chemical energy. The typical efficiency of photosynthesis in sugarcane ranges from 1 to 3%, which means that approximately 1–3% of the incident solar energy is converted into chemical energy through photosynthesis in sugarcane. The efficiency of solar energy utilization was positively correlated with solar radiation. However, it is important to note that this efficiency can be influenced by factors such as leaf area index, temperature, light intensity, water availability, nutrient status, and carbon dioxide concentration [42]. Sugarcane is a C4 plant, which means it has an efficient carbon fixation pathway that enables it to perform photosynthesis more efficiently under high light and temperature conditions. This C4 pathway allows sugarcane to effectively capture and utilize carbon dioxide, resulting in higher photosynthetic rates and biomass production compared to C3 plants. Promising results were observed in terms of solar energy utilization among the energy canes. Specifically, SBIEC14006 and SBIEC11001 (type-II), SBIEC11008 and SBIEC1100 (type-I) demonstrated higher solar energy utilization. These findings highlight the potential of both type-II and type-I energy canes for effectively harnessing solar energy and converting it into usable biomass for bioenergy production [43].

Cane growth and quality parameters play a crucial role in the success and productivity of sugarcane production [44, 45]. Monitoring and understanding these parameters are essential for optimizing cultivation practices, maximizing yield, and ensuring the production of high-quality sugarcane. Quality parameters such as sucrose content, fiber content, and juice purity significantly impact the commercial value and processing efficiency of sugarcane. In the current study, the clones were classified into two categories, i.e., the clones that exhibited juice brix levels above 15% and cane fiber content above 20% as Type I energy canes. Type-I energy clones with comparable pol% in juice were observed in SBIEC 14003, SBIEC 11005, SBIEC 13008 and SBIEC 13007. The high juice brix makes them suitable for direct fermentation in distilleries to produce alcohol, while the bagasse, the residue left after juice extraction, can be utilized in cogeneration units to generate electricity [46]. On the other hand, Type II energy canes are characterized by a cane fiber content exceeding 25% and a juice brix level below 15%. These energy canes can be harvested as whole canes, including trash and tops, which can be directly fed into boilers for electricity production [23]. By distinguishing between Type I and Type II energy canes based on their fiber content and juice brix, one can optimize their utilization for specific industrial applications, such as alcohol production and electricity generation [47]. In this work, it was identified that certain energy cane varieties, namely, SBIEC14006, SBIEC11004, and SBIEC11001, exhibit high fiber content. The high fiber percentage in energy canes offers numerous advantages and opportunities for various industries, including their utilization as an excellent source of feedstock for biofuel production, applications in the pulp and paper industry, textile, and apparel industry, and as animal feed demonstrates its versatility and economic viability. Fibrous biomass can be converted into biofuels such as cellulosic ethanol, biodiesel, and biogas, reducing dependence on fossil fuels and mitigating environmental impact [48, 49]. Therefore, focusing on the cultivation and utilization of high-fiber energy canes further contributes to the development of greener and cleaner energy [50].

The harvest index is a crucial parameter in breeding and selection programs [51]. Varieties with higher HIs are desirable because they indicate better partitioning of resources toward the harvested product [52, 53]. In this study, a higher harvest index (HI) in energy cane varieties was observed and compared to sugarcane. Type-I energy canes, specifically SBIEC 14002, displayed the highest harvest index of 0.81, followed by SBIEC 11002 with an HI of 0.74. Among type-II energy canes, SBIEC 14003 exhibited the highest HI of 0.84, while SBIEC 14006 recorded an HI of 0.61. The higher harvest index in energy canes highlights their potential as a more efficient source of biomass for energy production compared to traditional sugarcane varieties [54–56]. In conclusion, the harvest index plays a vital role in sugarcane cultivation by providing insights into yield potential, guiding breeding efforts, optimizing resource management, impacting economic viability, and improving crop efficiency. Monitoring and improving the harvest index can contribute to sustainable and productive sugarcane production systems.

## Conclusions

Energy canes had demonstrated commendable resilience, allowing it to thrive in diverse climatic conditions and environments. Its robust growth and adaptability enable it to withstand various stress factors, including drought, pests, and diseases, ensuring reliable and sustainable biomass production. The selection criteria for energy canes differ significantly from those used for commercial sugarcane varieties. While choosing energy canes, the focus should be primarily on traits related to biomass production, fiber content, and energy potential, rather than traits associated with sugar production. In this study, we have identified that the high biomass, high fiber content and higher energy utilization efficient canes, can be viable options to sustain the growing demand of biomass as feedstock to the sugar industries, which in turn can additionally help in replacing traditional coal-based industries.

### Electronic supplementary material

Below is the link to the electronic supplementary material.

Supplementary Material 1

Supplementary Material 2

Supplementary Material 3

## Data Availability

Data related to this publication are included in the manuscript and rest of the data is given in supplementary files.

## Abbreviations

FBM: fresh biomass yield
HI: harvest index
NMC: number of millable canes
SCW: single cane weight
DCW: dry cane weight
TDM: total dry weight
DTP: drought tolerance potential
