# Supplementary figures and images for: Biomass and energy potential of Erianthus arundinaceus and Saccharum spontaneum-derived novel sugarcane hybrids in rainfed environments
Source: BMC Plant Biol. 2024 Mar 19;24:198. doi: 10.1186/s12870-024-04885-0 (PMC10949791; doi:10.1186/s12870-024-04885-0)

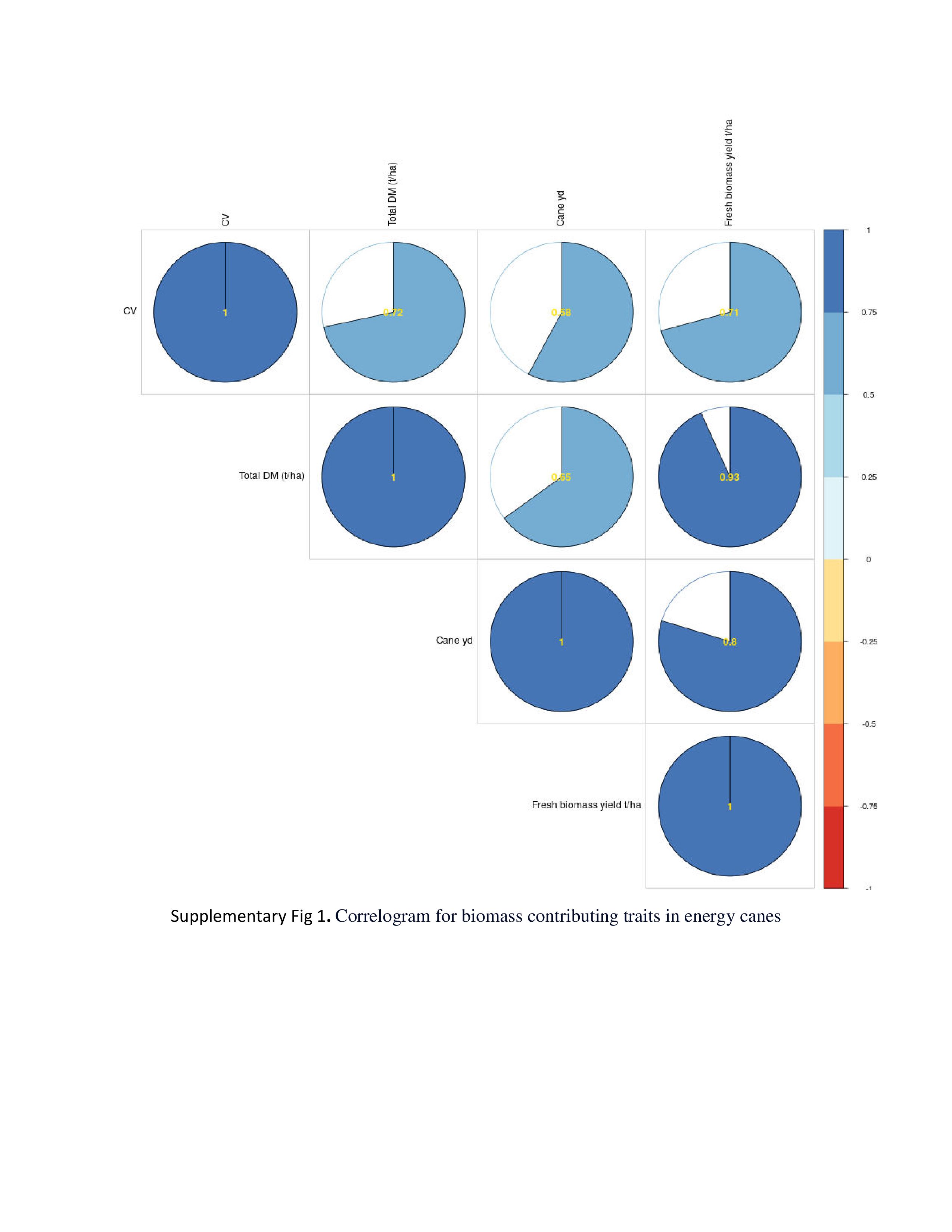

Supplement: Supplementary file 1 — Supplementary Material 1 [file 12870_2024_4885_MOESM1_ESM.jpg]
