# Supplementary material for: Biomass and energy potential of Erianthus arundinaceus and Saccharum spontaneum-derived novel sugarcane hybrids in rainfed environments
Source: BMC Plant Biol. 2024 Mar 19;24:198. doi: 10.1186/s12870-024-04885-0 (PMC10949791; doi:10.1186/s12870-024-04885-0)
Supplement: Supplementary file 2 — Supplementary Material 2 [file 12870_2024_4885_MOESM2_ESM.docx]

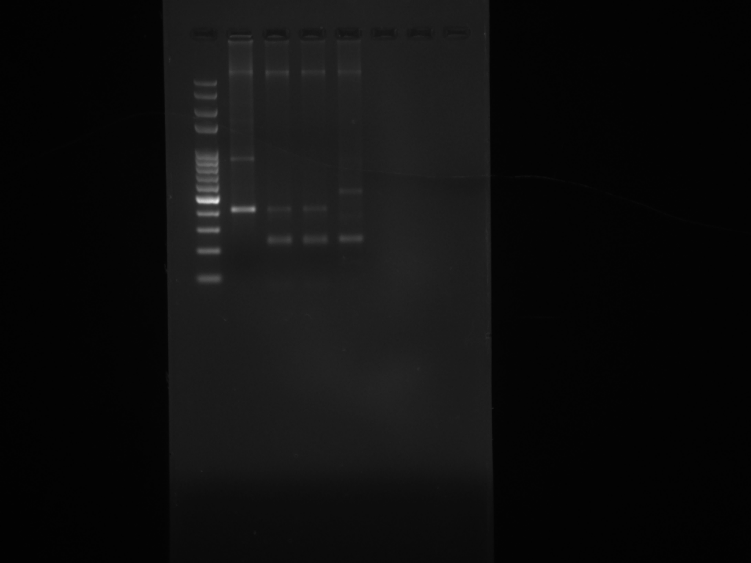


1 2 3 4 5

**500 bp**

**Supplementary Figure 2.** **Confirmation of *Erianthus* and *Saccharum* specific band in energy** clones- PCR amplification for 5s rDNA of energy canes and Commercial cultivars; Lane1 - 100 bP, Lane 2 – *Erianthus arundinaceus*, Lane 3 –Intergeneric hybrid, Lane 4 -Intergeneric hybrid and Lane 5-Co86032, *Erianthus* specific band – Orange arrow, *Saccharum* specific band – White arrow
