# Supplementary material for: Biomass and energy potential of Erianthus arundinaceus and Saccharum spontaneum-derived novel sugarcane hybrids in rainfed environments
Source: BMC Plant Biol. 2024 Mar 19;24:198. doi: 10.1186/s12870-024-04885-0 (PMC10949791; doi:10.1186/s12870-024-04885-0)
Supplement: Supplementary file 3 — Supplementary Material 3 [file 12870_2024_4885_MOESM3_ESM.docx]

|  | **Brix %** | | **Pol%** | | **Purity%** | | **Juice extract%** | |
| --- | --- | --- | --- | --- | --- | --- | --- | --- |
| Clones name | Plant | Ratoon | Plant | Ratoon | Plant | Ratoon | Plant | Ratoon |
| **Type-I Energy canes** | | | | | | | | |
| SBI-EC 11002 | 16.26 | 13.78 | 13.71 | 13.16 | 84.33 | 95.50 | 31.30 | 31.38 |
| SBI-EC 11003 | 17.64 | 13.31 | 15.84 | 12.28 | 89.80 | 92.30 | 27.46 | 23.08 |
| SBI-EC 11005 | 17.44 | 16.63 | 16.09 | 15.45 | 92.25 | 92.93 | 19.72 | 16.31 |
| SBI-EC 11008 | 13.40 | 12.87 | 11.28 | 11.09 | 84.16 | 86.17 | 19.03 | 19.89 |
| SBI-EC 11009 | 14.69 | 14.87 | 12.33 | 12.85 | 83.92 | 86.42 | 26.80 | 23.42 |
| SBI-EC 13001 | 17.58 | 14.17 | 15.82 | 12.06 | 89.96 | 85.11 | 15.83 | 12.56 |
| SBI-EC 13002 | 17.14 | 17.88 | 14.85 | 14.97 | 86.66 | 83.75 | 34.88 | 32.23 |
| SBI-EC 13005 | 17.47 | 14.89 | 15.90 | 12.85 | 91.00 | 86.30 | 30.39 | 29.99 |
| SBI-EC 13007 | 18.49 | 16.17 | 16.40 | 14.25 | 88.72 | 88.12 | 39.12 | 31.05 |
| SBI-EC 13008 | 17.71 | 17.12 | 16.50 | 16.24 | 93.16 | 94.86 | 33.79 | 28.15 |
| SBI-EC 13009 | 19.29 | 15.75 | 17.03 | 13.82 | 88.27 | 87.75 | 23.37 | 23.25 |
| SBI-EC 13010 | 15.82 | 14.87 | 13.62 | 12.94 | 86.11 | 87.02 | 38.41 | 39.15 |
| SBI-EC 14002 | 16.00 | 14.13 | 13.79 | 12.10 | 86.21 | 85.66 | 49.54 | 46.95 |
| SBI-EC 14003 | 19.11 | 18.22 | 17.12 | 16.96 | 89.55 | 93.06 | 32.63 | 32.18 |
| Mean | 17.00 | 15.33 | 15.02 | 13.64 | 88.15 | 88.92 | 30.16 | 27.83 |
| **Type-II Energy Canes** | | | | | | | | |
| SBI-EC 11006 | 6.73 | 6.44 | 3.27 | 3.83 | 48.65 | 59.44 | 26.04 | 25.83 |
| SBI-EC 11004 | 10.61 | 10.03 | 8.17 | 8.31 | 70.33 | 71.45 | 14.59 | 12.57 |
| SBI-EC 11001 | 8.73 | 8.39 | 2.76 | 2.79 | 31.66 | 33.19 | 19.20 | 15.33 |
| SBI-EC 14006 | 10.39 | 8.81 | 7.89 | 5.39 | 75.94 | 68.95 | 24.14 | 21.02 |
| Mean | 9.37 | 8.57 | 5.52 | 5.08 | 56.64 | 58.26 | 20.99 | 18.69 |
| **Checks Varieties** | | | | | | | | |
| **Co 0238** | 19.23 | 18.45 | 18.00 | 16.57 | 93.60 | 89.81 | 56.60 | 49.33 |
| **CoS 767** | 18.27 | 17.15 | 15.57 | 15.37 | 85.23 | 89.59 | 49.00 | 44.11 |
| Mean | 18.75 | 18.30 | 16.79 | 16.02 | 89.42 | 87.51 | 52.80 | 46.72 |
| **Grand Mean** | 15.65 | 12.69 | 13.30 | 8.58 | 84.96 | 67.58 | 30.59 | 27.89 |
| **LSD @ 5%** | 0.74 | 3.56 | 0.89 | 6.89 | 5.98 | 6.03 | 9.72 | 8.24 |

**Supplementary Table 1. Relative quality traits of Type-I and Type-II Energy canes along with checks varieties**
